# Sleep-related rhythmic movement disorder in children: a mini-review

**DOI:** 10.3389/fneur.2023.1165130

**Published:** 2023-05-15

**Authors:** Nhi Lam, Montida Veeravigrom

**Affiliations:** ^1^Sleep Medicine Program, Department of Pulmonary Medicine, University of Chicago, Chicago, IL, United States; ^2^Section of Child Neurology and Pediatric Sleep Medicine, Department of Pediatrics, University of Chicago, Chicago, IL, United States

**Keywords:** rhythmic movement disorder, children, video-polysomnography, seizure, sleep

## Abstract

Sleep-related rhythmic movement disorder (SRRMD) occurs in both infants and children. This disorder rarely occurs or persists in adolescence or adulthood. Rhythmic movement during sleep in children is often asymptomatic and considered a benign condition. It is classified as SRRMD when movement significantly disrupts sleep, results in daytime functional impairment, or causes self-inflicted body injury. Several studies have demonstrated that SRRMD occurs in all sleep stages. Few studies have investigated rhythmic movement disorder (RMD) in children. SRRMD is a clinical diagnosis supported by home video recordings. When the clinical history is insufficient to provide a definitive diagnosis of SRRMD, and other sleep-related conditions or seizure disorders are suspected, video-polysomnography is indicated. There are currently no clinical guidelines for treating SRRMD.

## Introduction

Sleep-related rhythmic movement disorder (SRRMD) occurs in both infants and children. This disorder rarely occurs or persists in adolescents or adults (1). The etiology of persistent SRRMD until late childhood and adulthood is multifactorial. Several studies have reported a bidirectional relationship between attention-deficit hyperactivity disorder (ADHD) and SRRMD (2–5). Rhythmic movement during sleep in children is often asymptomatic and is considered a benign condition (1). It is classified as SRRMD when movement significantly disrupts sleep, results in daytime function impairment, or causes self-inflicted body injury (6). There have been case reports of SRRMD causing alopecia (7), large skull defects (8), cataract (9), subdural hemorrhage (10), and Hirayama disease (11). Initially, SRRMD was classified as a sleep–wake transition disorder (12).

Several studies have demonstrated that SRRMD occurs most frequently during sleep–wake transition and non-rapid eye movement (NREM) 1 and NREM 2 sleep (2, 4, 13, 14). The subtypes of SRRMD include head banging (jactatio capitis nocturna), head rolling, body rocking, body rolling, and rarely, leg rolling or leg banging. Patients usually present with a single subtype of SRRMD. However, some patients experience two to three subtypes of SRRMD throughout the night (13–15). Little research has been conducted on RMD in children over the past 118 years since the first article was published in 1905 (16). We conducted an extensive literature review through the PubMed database with the search of “Sleep related rhythmic movement disorder,” “Rhythmic movement disorder,” “Sleep related movement disorder AND/Or Children.” Most research articles were case reports, case series, or review articles. However, over the past 5 years, original research articles have been published on the prevalence, maternal characteristics, and behavioral/emotional problems (1, 15, 17). One article examined the prevalence and clinical features of children with DS in a specific population (18).

## Prevalence

Sleep-related rhythmic movement disorder (SRRMD) is seen at varying ages but becomes less prevalent with increasing age. The prevalence of rhythmic head or body movements in normally developing infants is high at 9 months of age (59–67%), decreasing to 33% by 18 months and dropping to 6% by 5 years of age (6, 19). A retrospective parental report on body rocking involving 813 patients showed a prevalence of 15.3% at the ages of 3–10 years, which decreased to 3.1% at ages 11–13 years (20). In 2001, a single-item questionnaire about rocking or swaying back and forth before falling asleep was administered to 1,407 school-age children (6.2–10.9 years); it demonstrated a prevalence of 8.3% (21). In 2007, a longitudinal follow-up study of 1,058 children in Quebec, Canada, showed a prevalence of body rocking and head banging of 5.5 and 2% in children 2.5 and 6 years old, respectively (22). Previous prevalence studies used only questionnaire information from children or parents, and the research question did not specifically address the ICSD-3 criteria for SRRMD.

Recent epidemiological research in a large population in 2019 showed a lower prevalence of SRRMD in infants and toddlers, with a maximum prevalence of 2.87% (all potential cases from the screening questionnaires) and a minimum prevalence of 0.34% (only objectively confirmed cases by home videosomnography) (15). SRRMD rarely occurs in adolescence and adulthood but can persist in children with an autism spectrum disorder or developmental delay into adulthood (23). SRRMD is also more common in children with Down syndrome (up to 15%) and is associated with poor sleep quality (18). The prevalence is common in male patients, with a ratio of 5:3 when confirmed with home videosomnography (15). Meanwhile, other studies have reported no significant difference between the sexes (19, 21). Body rocking was the most common SRRMD subtype (43%), followed by head rolling (24%) and head banging (22%) (6). Rolling movements are associated with REM sleep, and rocking movements are observed during superficial NREM sleep (2).

## Diagnosis

The International Classification of Sleep Disorders (third edition) (ICSD-3) diagnostic criteria for SRRMD are as follows: (1) repetitive, stereotyped, and rhythmic motor behaviors involving large muscle groups; (2) movement is predominantly sleep-related; (3) behaviors result in significant complaints of interference with normal sleep, significant impairment in daytime function, or self-inflicted bodily injury; and (4) rhythmic movements are not better explained by another movement disorder or epilepsy (6).

The ICSD-1 classifies SRRMD as a sleep–wake transition disorder (12); it was reclassified as a sleep-related movement disorder in the ICSD-2 (24). The ICSD-3 highlights that SRRMD persists into adulthood (6). SRRMD is a clinical diagnosis supported by home video recordings (25). Parental reports may be unreliable for sleep disorders because the symptoms cannot be observed. Video-polysomnography is indicated when the clinical history is insufficient to provide a definitive diagnosis of SRRMD, and other sleep-related conditions or seizure disorders are suspected (26).

The American Academy of Sleep Medicine (AASM) described RMD characteristics as a minimum of four rhythmic movements with frequencies between 0.5 and 2.0 Hz and amplitude two times the background electromyography (EMG) activity (27). Gogo et al. used three nights of home videosomnography to confirm parental reports of SRRMD. Their findings showed unreliable parental reports (rhythmic movements confirmed by video 53%). Thus, objective confirmation of the diagnosis is important. However, there are no standardized measures for using home videos to diagnose SRRMD (15). It is time-consuming to manually analyze video data from polysomnography or perform a three-night home videosomnography. Recent research has shown promising data for using novel automatic three-dimensional (3D) analysis to diagnose SRRMD (28).

## Differential diagnosis

Several other repetitive movements can mimic SRRMD (Table 1), including sleep-related bruxism, thumb sucking, rhythmic sucking of the thumb or lips, and hypnagogic foot tremors (6). Children with autism can exhibit repetitive behavior; however, movement is usually observed while the child is awake. SRRMD occurs most frequently during waking and NREM 1 and 2 sleep (2, 4, 13, 14). Unlike REM sleep behavior disorders, SRRMD is more monophasic and not goal-directed (29). SRRMD can occur in the background of normal REM sleep with atonia.

**Table 1:** Differential diagnosis of sleep-related rhythmic movement disorder (SRRMD).

Nocturnal seizure: Sleep-related hyper motor epilepsy (SHE), Benign epilepsy with centrotemporal spikes, Benign occipital epilepsy.
Parasomnia
Other sleep-related movement disorders, such as hypnagogic foot tremor or sleep-related bruxism
Thumb sucking
Motor stereotypies in neurodevelopmental disability

Another important differential diagnosis of SRRMD is the occurrence of seizures during sleep, previously called nocturnal seizures. However, with recent evidence, the seizure in this epilepsy syndrome has occurred predominantly during sleep. Sleep-related hypermotor epilepsy (SHE) may manifest as brief stereotypical movements during sleep or after arousal from sleep. SHE is typically observed in the NREM stages N1 and N2 (6) and lasts 1–2 min (30). Ictal and interictal electroencephalograms (EEGs) are usually normal when the epileptiform focus is localized in deeper parts of the brain. However, SHE has a later age of onset, generally between 10 and 16 years (31). Sleep-related seizures, such as benign epilepsy with centrotemporal spikes and benign occipital epilepsy, have also been observed in other types of common epilepsy in children. Other manifestations can help distinguish seizures from SRRMD. They include tonic–clonic movements, incontinence, tongue biting, vomiting, risk factors for epilepsy, and inability to distract or follow commands to stop movement (32).

Sleep-related rhythmic movement disorder has been observed in normally developing children. It also occurs more frequently in children with neurodevelopmental disabilities, such as autism, Smith–Magenis syndrome, tuberous sclerosis, and Down syndrome (1, 15). SRRMD is also associated with other sleep disorders, such as obstructive sleep apneas (33), night terror (34), REM sleep behavior disorder (35, 36), periodic limb movement disorder, and insomnia (1, 32, 37). There is a bidirectional association between persistent SRRMD and ADHD (2, 4). Affective disorders, such as depression or anxiety, are comorbidities associated with SRRMD in children (1, 2, 13). SSRMD and comorbidities are listed in Table 2. Primary (idiopathic) SRRMD is rare, occurring in only 8% of the study population (1).

**Table 2:** Comorbidity with SRRMD.

Obstructive sleep apnea
Restless leg syndrome/periodic limb movement disorder
Night terror/parasomnia/REM sleep behavior disorder
Insomnia
Attention deficit hyperactivity disorder/learning disability
Neurodevelopmental disorder
Affective disorders such as depression and anxiety disorder

## Pathophysiology

The pathophysiology of this condition remains unclear. Several hypotheses regarding the pathophysiology of SRRMD have been proposed.

### The hypothesis of normal developmental variant

Rhythmic movements during sleep are physiological and occur in early infancy, coinciding with milestones of psychomotor development. This hypothesis demonstrates that rhythmic movements including head or whole body shaking stimulate the vestibular system, which positively affects motor development in infants (38).

### The hypothesis of self-soothing learning behavior

Initially, SRRMD was thought to occur only during wakefulness or sleep–wake transitions. SRRMD was believed to be a conditioned behavior to induce sleep at the beginning and the middle of the night (39). However, polysomnography has shown that SRRMD occurs in all sleep stages, including REM sleep. It is thought that rocking movements mimic maternal movement, heartbeat, and respiration *in utero* (32). Laganiere et al. showed that SRRMD is associated with lower maternal sensitivity, which is measured by observing the accuracy and appropriateness of the mother's response to the infant's needs (17).

### The hypothesis of disordered arousal

Sleep-related rhythmic movement disorder has been reported with sleep fragmentation and instability of NREM sleep, as demonstrated in the case report of a 9-year-old with SRRMD and a cyclic alternating pattern (40). The arousal pathway originates in the brainstem and involves the thalamocortical reticular system.

### The hypothesis of a central pattern motor generator

Sleep-related rhythmic movement disorder is caused by abnormalities of inhibitory control in the central motor pattern generator (association with impaired GABA release) (6, 33), as evidenced in neurodevelopmental disorders, which also involve impaired GABA release. This hypothesis may explain the high prevalence of SRRMD in neurodevelopmental disorders and the successful treatment with GABAergic medication.

### Genetic predisposition

There have been case reports of monozygotic triplets (41) and monozygotic twins (42). A case report showed multigenerational families with SRRMD and insomnia (37). However, the genetic basis is not fully understood.

## Treatment

Rhythmic movements during sleep in infants and children do not require pharmacological treatment. SRRMD is a rare disorder that affects children and their families; therefore, there are currently no clinical guidelines for SRRMD treatment due to a lack of randomized controlled trials which focus on pharmacological and behavioral treatment. There are clinical reports in the literature on hypnosis (43), benzodiazepines (44), melatonin (45), sleep restriction (46), and cognitive behavioral therapy (47). Clonazepam was the first reported successful treatment in adults with RMD (48). Moreover, there have been case reports of successful treatment with clonazepam in children with fragile X (44). Alves et al., on the other hand, report no clinical improvement with the use of clonazepam and midazolam but reported good clinical outcomes using imipramine (49). Metin et al. reported improvement in head banging in an 8-year-old girl with melatonin doses up to 9 mg, but complete remission was not achieved (45). Behavioral approaches including immediate feedback, reward system, and aversion therapy described in a small number of case studies showed some improvement (47). A combination of sleep restriction and hypnotics using chloral hydrate showed complete or almost complete resolution of SRRMD in six children with RMD. However, the sample size was small, and the results have not been replicated in a randomized controlled trial (46). SRRMD is associated with other sleep disorders, such as obstructive sleep apnea or periodic limb movement disorder. Treating sleep disorders with methods such as adenotonsillectomy or CPAP can also resolve SRRMD (33, 50, 51). Other interventions for SRRMD focus on safety and injury prevention and include bed padding, removing the bed from the wall, using bed rails, and confirming the stability of the patient's bed [(32), 53].

## Discussion

Sleep-related rhythmic movement disorder is common among children; in some rare cases, patients may start experiencing persistent SRRMD during adolescence and adulthood. It is a highly comorbid condition that warrants diagnosis and treatment. Therefore, further evaluation of other sleep disorders and related comorbidities is necessary.

## Author contributions

Both authors listed have made a substantial, direct, and intellectual contribution to the work and approved it for publication.

## References

[1] Laganière C Pennestri MH Rassu AL Barateau L Chenini S Evangelista E. Disturbed nighttime sleep in children and adults with rhythmic movement disorder. Sleep. (2020) 43:zsaa105. 10.1093/sleep/zsaa105 32459316

[2] Prihodova I Skibova J Nevsimalova S. Sleep-related rhythmic movements, and rhythmic movements disorder beyond early childhood. Sleep Med. (2019) 64:112–5. 10.1016/j.sleep.2019.05.021 31683092

[3] Dyken ME Lin-Dyken DC Yamada T. Diagnosing rhythmic movement disorder with video-polysomnography. Pediatr Neurol. (1997) 16:37–41. 10.1016/S0887-8994(96)00259-7 9044399

[4] Stepanova I Nevsimalova S Hanusova J. Rhythmic movement disorder in sleep persisting into childhood and adulthood. Sleep. (2005) 28:851–7. 10.1093/sleep/28.7.851 16124665

[5] Silvestri R Gagliano A Aricò I Calarese T Cedro C Bruni O. Sleep disorders in children with attention-deficit/hyperactivity disorder (ADHD) recorded overnight by video-polysomnography. Sleep Med. (2009) 10:1132–8. 10.1016/j.sleep.2009.04.003 19527942

[6] American Academy of Sleep Medicine. International Classification of Sleep Disorders. 3rd ed. Darien, IL: American Academy of Sleep Medicine (2014).

[7] Oska S Zarbo A Jahnke MN. Sleep-related rhythmic movement disorder: a case report of head banging alopecia. Pediatr Dermatol. (2020) 37:350–1. 10.1111/pde.14085 31930572

[8] Stuck KJ Hernandez RJ. Large skull defect in a headbanger. Pediatr Radiol. (1979) 8:257–8. 10.1007/BF00974046 514683

[9] Bemporad JR Sours JA Spalter HF. Cataracts following chronic headbanging: a report of two cases. Am J Psychiatry. (1968) 125:245–9. 10.1176/ajp.125.2.245 5662521

[10] Mackenzie JM. ‘Headbanging' and fatal subdural haemorrhage. Lancet. (1991) 338:1457–8. 10.1016/0140-6736(91)92757-S 1683440

[11] Jeannet PY Kuntzer T Deonna T Roulet-Perez E. Hirayama disease associated with a severe rhythmic movement disorder involving neck flexions. Neurology. (2005) 64:1478–9. 10.1212/01.WNL.0000158678.17161.1B 15851753

[12] Thorpy MJ the Diagnostic Classification Steering Committee. The International Classification of Sleep Disorders: Diagnostic and Coding Manual. 2nd ed. Rochester, NY: American Sleep Disorders Association, MN (1990). p. 151–4.

[13] Mayer G Wilde-Frenz J Kurella B. Sleep related rhythmic movement disorder revisited. J Sleep Res. (2007) 16:110–6. 10.1111/j.1365-2869.2007.00577.x 17309770

[14] Kohyama J Matsukura F Kimura K Tachibana N. Rhythmic movement disorder: polysomnographic study and summary of reported cases. Brain Dev. (2002) 24:33–8. 10.1016/S0387-7604(01)00393-X 11751023

[15] Gogo E van Sluijs RM Cheung T Gaskell C Jones L Alwan NA. Objectively confirmed prevalence of sleep-related rhythmic movement disorder in pre-school children. Sleep Med. (2019) 53:16–21. 10.1016/j.sleep.2018.08.021 30384137

[16] Zappert J Bei Kindern UNK. Jactatio capitis nocturna. Jahrb f Kinderheklkd. (1905) 62:70–83.

[17] Laganière C Gaudreau H Pokhvisneva I Atkinson L Meaney M Pennestri MH. Maternal characteristics and behavioural/emotional problems in preschoolers: How they relate to sleep rhythmic movements at sleep onset. J Sleep Res. (2019) 28:e12707. 10.1111/jsr.12707 29873138

[18] Kose C Wood I Gwyther A Basnet S Gaskell C Gringras P. Sleep-related rhythmic movement disorder in young children with Down syndrome: prevalence and clinical features. Brain Sci. (2021) 11:1326. 10.3390/brainsci11101326 34679391 PMC8533778

[19] Klackenberg G. Rhythmic movements in infancy and early childhood. Acta Paediatr Scand. (1971) 224:74.e83.

[20] Laberge L Tremblay RE Vitaro F Montplaisir J. Development of parasomnias from childhood to early adolescence. Pediatrics. (2000) 106:67–74. 10.1542/peds.106.1.67 10878151

[21] Nevéus T Cnattingius S Olsson U Hetta J. Sleep habits and sleep problems among a community sample of schoolchildren. Acta Paediatr. (2001) 90:1450–5. 10.1111/j.1651-2227.2001.tb01612.x 11853345

[22] Petit D Touchette E Tremblay RE Boivin M Montplaisir J. Dyssomnias and parasomnias in early childhood. Pediatrics. (2007) 119:e1016–25. 10.1542/peds.2006-2132 17438080

[23] Vetrugno R Montagna P. Sleep-to-wake transition movement disorders. Sleep Med. (2011) 12 Suppl 2:S11–6. 10.1016/j.sleep.2011.10.005 22136891

[24] American Academy of Sleep Medicine. The International Classification of Sleep Disorders, Revised. Chicago, IL: American Academy of Sleep Medicine (2005). 297 p.

[25] Ferri R Fulda S Chapter 168. Recording and scoring sleep-related movements. In:Kryger M Roth T Dement WC, editors. Principles and Practice of Sleep Medicine. 6th ed. Philadelphia, PA: Elsevier (2017). p. 1633–50.e6.

[26] Singer HS Mink JW Gilbert DL Jankovic J Chapter 19. Movements that occur. 2nd ed. In:Harvey S. Singer Jonathan W. Mink Donald L, editors. Movement Disorders in Childhood. Sandiego, CA: Academic Press (2016). p. 427–51.

[27] Berry RB Quan SF Abreu AR Bibbs ML DelRosso L Harding SM et al.; for the American Academy of Sleep Medicine. The AASM Manual for the Scoring of Sleep and Associated Events: Rules, Terminology and Technical Specification. version 2.6. Darien, IL: American Academy of Sleep Medicine (2020).

[28] Gall M Kohn B Wiesmeyr C van Sluijs RM Wilhelm E Rondei Q. A novel approach to assess sleep-related rhythmic movement disorder in children using automatic 3D analysis. Front Psychiatry. (2019) 10:709. 10.3389/fpsyt.2019.00709 31681030 PMC6806394

[29] Salas R Gulyani S Kwan A Gamaldo C. Sleep related movement disorders and their unique motor manifestations. In:Kryger M Roth T Dement WC, editors. Principles and Practice of Sleep Medicine. 6th ed. Philadelphia, PA: Elsevier (2017). p. 1020–9.

[30] Rosen GM. Disorders of arousal. In:Medicine SSH Ferber R Kryger MH, editors. Principles and Practice of Pediatric Sleep. Philadelphia, PA: Elsevier Saunders (2014). 356 p.

[31] Tinuper P Bisulli F Cross JH Hesdorffer D Kahane P Nobili L. Definition and diagnostic criteria of sleep-related hypermotor epilepsy. Neurology. (2016) 86:1834–42. 10.1212/WNL.0000000000002666 27164717 PMC4862248

[32] Gwyther ARM Walters AS Hill CM. Rhythmic movement disorder in childhood: an integrative review. Sleep Med Rev. (2017) 35:62–75. 10.1016/j.smrv.2016.08.003 27884450

[33] Bishara J Mitacek R. Images: rhythmic movement disorder in a normal developing child with obstructive sleep apnea. J Clin Sleep Med. (2021) 17:2137–9. 10.5664/jcsm.9428 34032201 PMC8494084

[34] Merli E Ferri R DelRosso LM Mignani F Loddo G Traverso A. Sleep-related rhythmic movements and sleep terrors: a possible common neurophysiological background in a preschool boy. J Clin Sleep Med. (2019) 15:1849–52. 10.5664/jcsm.8098 31855169 PMC7099188

[35] Manni R Terzaghi M. Rhythmic movements in idiopathic REM sleep behavior disorder. Mov Disord. (2007) 22:1797–800. 10.1002/mds.21622 17580329

[36] Xu Z Anderson KN Shneerson JM. Association of idiopathic rapid eye movement sleep behavior disorder in an adult with persistent, childhood onset rhythmic movement disorder. J Clin Sleep Med. (2009) 5:374–5. 10.5664/jcsm.27551 19968018 PMC2725259

[37] Attarian H Ward N Schuman C. A multigenerational family with persistent sleep related rhythmic movement disorder (RMD) and insomnia. J Clin Sleep Med. (2009) 5:571–2. 10.5664/jcsm.27660 20465026 PMC2792975

[38] Manni R Terzaghi M. Rhythmic movements during sleep: a physiological and pathological profile. Neurol Sci. (2005) 26(Suppl. 3):s181–5. 10.1007/s10072-005-0484-8 16331393

[39] Sallustro F Atwell CW. Body rocking, head banging, and head rolling in normal children. J Pediatr. (1978) 93:704–8. 10.1016/S0022-3476(78)80922-6 309000

[40] Manni R Terzaghi M Sartori I Veggiotti P Parrino L. Rhythmic movement disorder and cyclic alternating pattern during sleep: a video-polysomnographic study in a 9-year-old boy. Mov Disord. (2004) 19:1186–90. 10.1002/mds.20133 15390015

[41] Hayward-Koennecke HK Werth E Valko PO Baumann CR Poryazova R. Sleep-related rhythmic movement disorder in triplets: evidence for genetic predisposition? J Clin Sleep Med. (2019) 15:157–8. 10.5664/jcsm.7594 30621834 PMC6329534

[42] Rosenberg C. Elimination of a rhythmic movement disorder with hypnosis–A case report. Sleep. (1995) 18:608–9. 10.1093/sleep/18.7.608 8552933

[43] Manni R Tartara A. Clonazepam treatment of rhythmic movement disorders. Sleep. (1997) 20:812. 10.1093/sleep/20.9.812 9406333

[44] Metin O Eynalli-Gok E Kuygun-Karci C Yolga-Tahiroglu A. The effectiveness of melatonin in Head Banging: a case report. Sleep Sci. (2019) 12:53–6. 10.5935/1984-0063.20190049 31105896 PMC6508939

[45] Etzioni T Katz N Hering E Ravid S Pillar G. Controlled sleep restriction for rhythmic movement disorder. J Pediatr. (2005) 147:393–5. 10.1016/j.jpeds.2005.06.045 16182683

[46] Sadeh A. Cognitive-behavioral treatment for childhood sleep disorders. Clin Psychol Rev. (2005) 25:612–28. 10.1016/j.cpr.2005.04.006 15979219

[47] Chisholm T Morehouse RL. Adult headbanging: sleep studies and treatment. Sleep. (1996) 19:343–6. 10.1093/sleep/19.4.343 8776793

[48] Alves RS Aloe F Silva AB Tavares SM. Jactatio capitis nocturna with persistence in adulthood. Case report. Arq Neuropsiquatr. (1998) 56:655–7. 10.1590/S0004-282X1998000400022 9850765

[49] Gharagozlou P Seyffert M Santos R Chokroverty S. Rhythmic movement disorder associated with respiratory arousals and improved by CPAP titration in a patient with restless legs syndrome and sleep apnea. Sleep Med. (2009) 10:501–3. 10.1016/j.sleep.2009.03.003 19362882

[50] Chirakalwasan N Hassan F Kaplish N Fetterolf J Chervin RD. Near resolution of sleep related rhythmic movement disorder after CPAP for OSA. Sleep Med. (2009) 10:497–500. 10.1016/j.sleep.2009.02.005 19324593

[51] Haywood PM Hill CM. Rhythmic movement disorder: Managing the child who head-bangs to get to sleep. Paediatr Child Health. (2012) 22:207–10. 10.1016/j.paed.2012.02.010

